# Characterization of *Chlamydomonas reinhardtii* phosphatidylglycerophosphate synthase in *Synechocystis* sp. PCC 6803

**DOI:** 10.3389/fmicb.2015.00842

**Published:** 2015-08-24

**Authors:** Chun-Hsien Hung, Kaichiro Endo, Koichi Kobayashi, Yuki Nakamura, Hajime Wada

**Affiliations:** ^1^Institute of Plant and Microbial Biology, Academia SinicaTaipei, Taiwan; ^2^Department of Life Sciences, Graduate School of Arts and Sciences, The University of TokyoTokyo, Japan; ^3^PRESTO, Japan Science and Technology AgencySaitama, Japan; ^4^CREST, Japan Science and Technology AgencySaitama, Japan

**Keywords:** *Chlamydomonas reinhardtii*, chloroplast, glycerolipid, phosphatidylglycerol, photosynthesis

## Abstract

Phosphatidylglycerol (PG) is an indispensable phospholipid class with photosynthetic function in plants and cyanobacteria. However, its biosynthesis in eukaryotic green microalgae is poorly studied. Here, we report the isolation and characterization of two homologs (CrPGP1 and CrPGP2) of phosphatidylglycerophosphate synthase (PGPS), the rate-limiting enzyme in PG biosynthesis, in *Chlamydomonas reinhardtii*. Heterologous complementation of *Synechocystis* sp. PCC 6803 *pgsA* mutant by *CrPGP1* and *CrPGP2* rescued the PG-dependent growth phenotype, but the PG level and its fatty acid composition were not fully rescued in the complemented strains. As well, oxygen evolution activity was not fully recovered, although electron transport activity of photosystem II was restored to the wild-type level. Gene expression study of *CrPGP1* and *CrPGP2* in nutrient-starved *C. reinhardtii* showed differential response to phosphorus and nitrogen deficiency. Taken together, these results highlight the distinct and overlapping function of PGPS in cyanobacteria and eukaryotic algae.

## Introduction

Photosynthetic membranes are highly specialized biological membranes that contain distinct yet conserved classes of polar glycerolipids MGDG, DGDG, SQDG, and PG among cyanobacteria, eukaryotic microalgae, and land plants (Omata and Murata, 1983; Dorne et al., 1990; Sakurai et al., 2006). Gene knockout affecting biosynthesis of these lipids causes severe photosynthetic defects in a cyanobacterium, *Synechocystis* sp. PCC 6803 (Mendiola-Morgenthaler et al., 1985; Hagio et al., 2000; Sato et al., 2000; Aoki et al., 2004; Awai et al., 2007; Sakurai et al., 2007b), the eukaryotic green microalga *Chlamydomonas reinhardtii* (Dubertret et al., 1994; Sato et al., 1995) and the seed plant *Arabidopsis thaliana* (Dörmann et al., 1995; Hagio et al., 2002; Babiychuk et al., 2003; Kelly et al., 2003; Yu and Benning, 2003; Kobayashi et al., 2007, 2015). Biosynthesis of these lipids may be critical for photosynthesis.

Except for variation in fatty acid composition, the structural features of these four lipid classes are highly similar; however, the biosynthetic pathways may be diverse among cyanobacteria, eukaryotic algae and higher plants. For example, the most abundant lipid class, MGDG, is synthesized in *Synechocystis* sp. PCC 6803 by two steps (Sato and Murata, 1982): first DAG is glucosylated with UDP-glucose to form MGlcDG by MGlcDG synthase (Awai et al., 2006), which is then isomerized to MGDG by an epimerase (Awai et al., 2014). However, in *A. thaliana* and other seed plants, MGDG is synthesized by one-step galactosylation with UDP-galactose by MGDG synthases (Shimojima et al., 1997). In contrast, DGDG is produced by the further galactosylation of MGDG by DGDG synthases in both *Synechocystis* sp. PCC 6803 (Awai et al., 2007) and *A. thaliana* (Kelly and Dormann, 2002; Kelly et al., 2003).

Phosphatidylglycerol is the only major phospholipid class present in the photosynthetic membrane. It has a distinct contribution to photosynthesis. Gene knockout study revealed the crucial role of PG biosynthesis: disruption of *pgsA*, encoding PGPS in *Synechocystis* sp. PCC 6803, affects cell growth and photosynthetic activity unless PG is supplemented exogenously (Hagio et al., 2000). Arabidopsis possesses two PGPS, PGP1 and PGP2; knocking out *PGP1* severely impairs chloroplast biogenesis but not mitochondrial function (Hagio et al., 2002; Babiychuk et al., 2003), and double knockout of *PGP1* and *PGP2* further reduces PG levels to a trace amount, and causing an embryonic-lethal phenotype (Tanoue et al., 2014).

Much less is known about the biosynthesis of photosynthetic membrane lipids in *C. reinhardtii*. However, a distinct feature of chloroplast lipid metabolism has been shown: an involvement of chloroplastic galactoglycerolipid lipase in triacylglycerol production in nitrogen-starved *C. reinhardtii* (Li et al., 2012). Because the possible contribution of chloroplastic glycerolipids in triacylglycerol production is uniquely observed in *C. reinhardtii*, dissecting the similarity and distinctiveness of chloroplastic lipid biosynthesis in *C. reinhardtii* with reference to *Synechocystis* sp. PCC 6803 and *A. thaliana* is important.

In this study, we isolated two *PGPS* genes in *C. reinhardtii*, *CrPGP1* and *CrPGP2*, and assessed the molecular function by transforming them into a *pgsA* mutant of *Synechocystis* sp. PCC 6803. Moreover, gene expression profiles were examined in *C. reinhardtii* under phosphorus or nitrogen-starved conditions. The result showed distinct and overlapping functions of PGPS between cyanobacteria and eukaryotic algae.

## Materials and Methods

### Strains and Growth Conditions

The wild-type and *pgsA* cells of *Synechocystis* sp. PCC 6803 were grown photoautotrophically at 30°C in BG-11 medium supplemented with 20 μM PG as described previously (Sakurai et al., 2003). Growth of cultures was monitored by determining optical density at 730 nm (OD_730_). Light was provided by fluorescent lamps with approximately 50–60 μmol photons m^-2^ s^-1^. *C. reinhardtii* strain CC-4351 was obtained from the Chlamydomonas Resource Center and transformed with empty pChlamiRNA2 plasmid (Molnar et al., 2009). Cells were photoheterotrophically grown in TAP medium (Gorman and Levine, 1965) at 22°C. Nutrient deficiency was induced by collecting the cells by centrifugation (5 min at 3000 × *g*), washed twice with the respective media and subsequently resuspended in TAP medium, TAP medium without nitrogen (TAP-N), or phosphorus (TAP-P) by omitting NH_4_Cl, or replacing potassium phosphate with 1.5 mM KCl, respectively (Quisel et al., 1996).

### Molecular Cloning

#### *CrPGP1* (Cre03.g162601)

A 937-bp fragment was amplified using cDNA synthesized from the total RNA of *C. reinhardtii* strain CC-503 (cw92 mt^+^) as the template with the primers CH223 and CH224 and cloned into pENTR/D_TOPO to construct pCH067. Then, the cloned fragment was amplified with the primers CH772 and CH773 and inserted into *Nde*I and *Hpa*I sites of pTCP2031V to construct pCH167.

#### *CrPGP2* (Cre02.g095106)

A 790-bp fragment was amplified using cDNA synthesized from the total RNA of *C. reinhardtii* strain CC-503 (cw92 mt^+^) as the template with the primers CH225 and CH226 and cloned into pENTR/D_TOPO to construct pCH068. Then, the cloned fragment was amplified with the primers CH774 and CH775 and inserted into *Nde*I and *Hpa*I sites of the pTCP2031V vector which was designed to incorporate a gene of interest at a neutral site (*slr2031*) with the *psbA2* (*slr1311*) promoter and a chloramphenicol-resistance cassette (Satoh et al., 2001). The resulting plasmid pCH160 was used to transform the *pgsA* mutant of *Synechocystis* sp. PCC 6803 by homologous recombination. The strains, plasmids, and oligonucleotide primers used in this study are described in Supplementary Tables 1–3, respectively.

### Complementation Assay of the *Synechocystis* sp. PCC 6803 *pgsA* Mutant by *CrPGP1* and *CrPGP2*

For culture on BG-11 agar plates, 5 μl of liquid culture was used with serial 10-fold dilution for spotting from left to right starting at OD_730_ 0.05 onto the plate with or without 20 μM PG. Plates were incubated photoautotrophically under 50∼60 μmol photons m^-2^ s^-1^ for 5 days at 30°C to observe the growth phenotype. For liquid culture, cells were photoautotrophically grown in BG-11 media. Initial growth was started at OD_730_ 0.1 by the stirring culture at 180 rpm, 50∼60 μmol photons m^-2^ s^-1^ at 30°C.

### Genotype Analysis

Genomic DNA was isolated from cells of the wild type, *pgsA CrPGP1* and *pgsA CrPGP2* of *Synechocystis* sp. PCC 6803. The primers CH784 and CH785 were used for PCR analysis of genetic background; a 1,141-bp fragment could be amplified from wild-type genomic DNA, whereas a 2,341-bp fragment could be amplified from genomic DNA of *pgsA CrPGP1* or *pgsA CrPGP2*. PCR analysis of the insertion at *slr2031* involved the primers for CH982 and CH1000, for an expected 575-bp fragment from the wild type, 2,938-bp fragment from *pgsA CrPGP1* and 2,791-bp fragment from *pgsA CrPGP2*.

### Lipid Analysis

Lipids were extracted from intact cells as described in (Bligh and Dyer, 1959) and analyzed previously (Nakamura et al., 2003).

### RNA Extraction and qRT-PCR Analysis

Total RNA from *Synechocystis* sp. PCC 6803 cells grown in BG-11 media by stirring culture at 180 rpm, 50∼60 μmol photons m^-2^ s^-1^ at 30°C was extracted using TRI reagent (Ambion) including DNase treatment and reverse-transcribed with SuperScript III (Invitrogen, Carlsbad, CA, USA) for cDNA synthesis. Quantitative RT-PCR involved the ABI 7500 Real Time PCR System (Applied Biosystems) with the oligonucleotide primers for *pgsA* (*slr 1522*; CH1028 and CH1029), *PGP1* (Cre03.g162601; CH919 and CH920), *PGP2* (Cre02.g095106; CH953 and CH954) and *RNase P subunit B* (*rnpB*; CH947 and CH948). Gene expression was normalized to that of *rnpB* (Yuzawa et al., 2014). Data were averaged by three technical replicates in the same run and three biological replicates in separate runs.

Total RNA extraction and cDNA synthesis from *C. reinhardtii* cells grown by stirring culture at 200 rpm, 50∼60 μmol photons m^-2^ s^-1^ at 22°C, follow the method as described above. Oligonucleotide primers used are: *Chlamydomonas G-protein beta subunit-like polypeptide* (*CBLP*; Cre06.g278222; CH1076 and CH1077), *PGP1* (Cre03.g162601; CH919 and CH920), *PGP2* (Cre02.g095106; CH953 and CH954), *monogalactosyldiacylglycerol synthase1* (*MGD1*; Cre13.g585301; CH890 and CH891), *digalactosyldiacylglycerol synthase1* (*DGD1*; Cre13.g583600; CH1060 and CH1061), *UDP-sulfoquinovose synthase* (*SQD1*; Cre16.g656400; CH1062 and CH1063), *sulfoquinovosyldiacylglycerol synthase* (*SQD2*; Cre01.g038550; CH1064 and CH1065), *nitrate reductase1* (*NIT1*; Cre09.g410950; CH1072 and CH1073) and *phosphorus starvation response protein* 1 (*PSR1*; Cre12.g495100; CH1066 and CH1067). Gene expression was normalized to that of *CBLP* (Schloss, 1990; Chang et al., 2005). Data were averaged by three technical replicates in the same run and two biological replicates in separate runs. Annotation of genes for the lipid metabolism is according to (Li-Beisson et al., 2015). The primers used are listed in Supplementary Table 3.

### Assay of Photosynthetic Parameters

Photosynthetic oxygen-evolving activity from H_2_O to CO_2_ of intact cells was measured with a Clark-type oxygen electrode (Hansatech Instruments, Kings Lynn, UK) as described in (Gombos et al., 1991). Light from an incandescent lamp through a red optical filter was used for all oxygen evolution measurements at the light intensity 900 μmol photons m^-2^ s^-1^. The Chl concentration of cells, determined by the method of Porra et al. (1989) with 100% methanol extraction, was adjusted to 5 μg mL^-1^.

For measurements of 77 K fluorescence emission spectra, intact cells were suspended in BG-11 at 10 μg Chl mL^-1^. Fluorescence emission spectra of intact cells under 435 or 600 nm excitation were recorded at 77 K with a spectrofluorometer (RF-5300PC; Shimadzu) as described (Sakurai et al., 2007a).

Measurements of relaxation of flash-induced Chl fluorescence yield were performed in intact cells (5 μg Chl mL^-1^) by profiling the spectra following single flash excitation with or without 10 μM DCMU (Vass et al., 1999). Samples were incubated in darkness for 10 min before DCMU was added at a final concentration of 10 μM. The levels of *F*_o_ and *F*_m_ were normalized.

For measurements of absorption spectra, cells were grown by stirring culture at 180 rpm, 50∼60 μmol photons m^-2^ s^-1^ at 30°C. Absorption spectra of pigments were determined directly in intact cell suspension (OD_730_≈0.1) by spectrophotometry (Beckman Coulter DU 800 Spectrophotometer). Spectra were normalized at 625 nm, the maximum absorption of phycobiliproteins, as described (Gombos et al., 2002).

## Results

### Isolation of Genes Encoding Putative PGPS from *C. reinhardtii*

**FIGURE 1:**
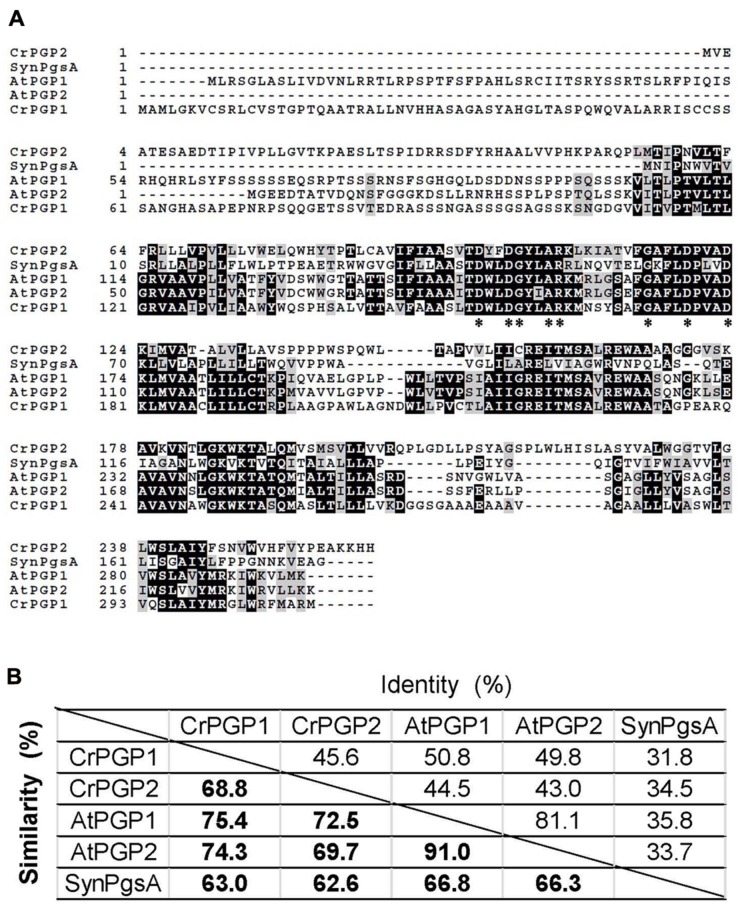
**Comparison of amino acid sequences of PGPs. (A)** Multiple amino acid sequence alignment of two PGPs of *Chlamydomonas reinhardtii* (CrPGP1 and CrPGP2) with reference to two PGPs of *Arabidopsis thaliana* (AtPGP1 and AtPGP2) and PgsA of *Synechocystis* sp. PCC 6803 (SynPgsA). Asterisks indicate the amino acid residues conserved in all sequences of proteins with the CDP-OH-P motif (PF01066.9). **(B)** The identity and similarity of amino acid sequences among PGPs aligned in **(A)**.

To isolate genes encoding functional PGPS for the biosynthesis of PG in *C. reinhardtii*, we performed a homology search with the amino acid sequence of AtPGP1 (At2g39290), which plays a major role in biosynthesis of PG and is essential for thylakoid membrane development in *A. thaliana* (Hagio et al., 2002; Xu et al., 2002; Babiychuk et al., 2003). Two genes were found as putative genes of PGPS in *C. reinhardtii* and named *CrPGP1* (Cre03.g162601) and *CrPGP2* (Cre02.g095106), encoding 32.1 and 28.7 kDa protein, respectively. We compared the amino acid sequence of CrPGP1 and CrPGP2 with characterized PGPs in *A. thaliana* (AtPGP1 and AtPGP2) (Hagio et al., 2002; Xu et al., 2002; Babiychuk et al., 2003) and *Synechosystis* sp. PCC 6803 (PgsA) (Hagio et al., 2000) by creating multiple amino acid sequence alignment (**Figure 1A**). As can be seen, the amino acid residues conserved in all sequences of proteins with the CDP-OH-P motif (PF01066.9) indicated by asterisks were conserved in CrPGP1 and CrPGP2, suggesting that these proteins are functional PGPs. Interestingly, CrPGP1 has longer N-terminal sequence than CrPGP2, as is found between AtPGP1 and AtPGP2. Next, we compared the identity and similarity of these PGPs (**Figure 1B**). The value ranges from 31.8 to 81.1% in identity and from 62.6 to 91.0% in similarity. The identity and similarity of PGPs were higher between *C. reinhardtii* and *A. thaliana* than CrPGP1 and CrPGP2, and those with *Synechocystis* PgsA were similar between *C. reinhardtii* and *A. thaliana*.

**FIGURE 2:**
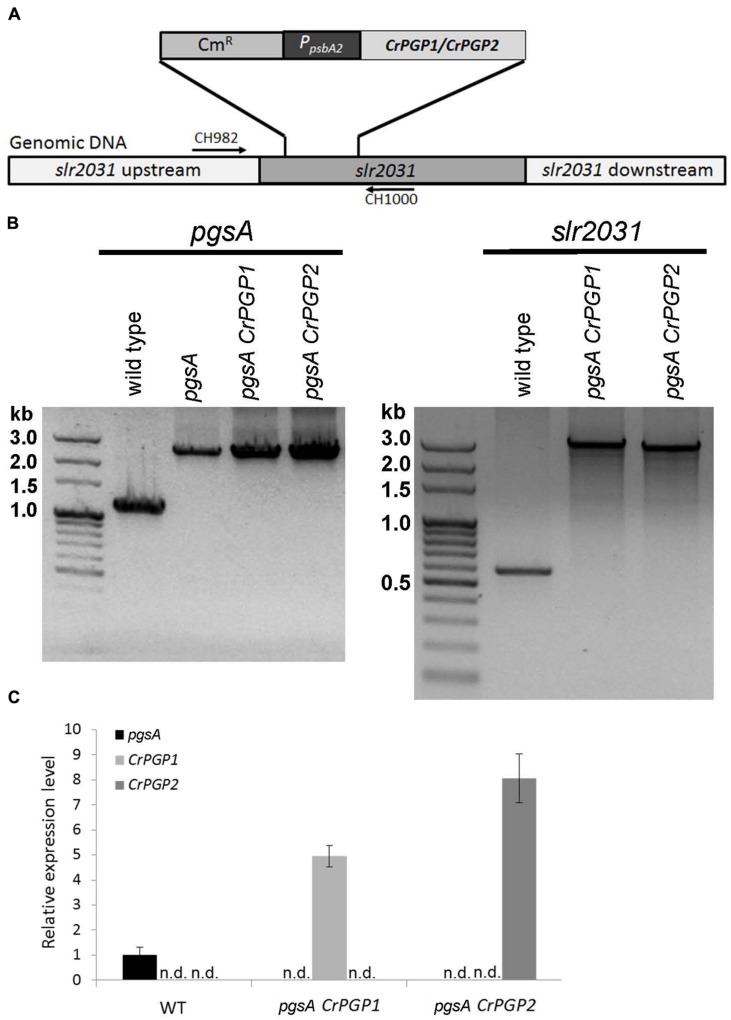
**Functional complementation of *Synechocystis sp*. PCC 6803 *pgsA* mutant with *PGP1* or *PGP2* of *Chlamydomonas reinhardtii*. (A)** Schematic illustration of homologous recombination of *CrPGPs* at *slr2031* locus. **(B)** PCR-based genotyping analysis of the transformants for *pgsA* (left panel) and *slr2031* (right panel). **(C)** Expression level of *CrPGP1* and *CrPGP2* in the wild type, *pgsA CrPGP1* and *pgsA CrPGP2* relative to that of *pgsA* in the wild type. Levels are normalized to that of *rnpB*. Data are mean ± SD from three biological replicates. n.d., not detected.

To examine whether these two genes encode functional PGPS, we cloned *CrPGP1* and *CrPGP2* under the control of the *psbA2* promoter and stably transformed them into a neutral genomic site (*slr2031*) (Satoh et al., 2001) of the *Synechocystis* sp. PCC 6803 *pgsA* mutant, which is defective in PGPS activity (Hagio et al., 2000) (**Figure 2A**). To confirm the successful homologous recombination of *CrPGP1* and *CrPGP2* into the neutral genomic site in the *pgsA* mutant, we performed PCR-based genotyping for both *pgs*A (*slr1522*) and *slr2031* (**Figure 2B**). Compared to the wild type, both *pgsA CrPGP1* and *pgsA CrPGP2* mutants maintained the *pgsA* background and the introduced genes were fully segregated at the *slr2031* locus by the homologous recombination. We further investigated whether *CrPGP1* and *CrPGP2* were expressed in *pgsA* by comparing mRNA levels of *CrPGP1* in *pgsA CrPGP1, CrPGP2* in *pgsA CrPGP2*, and *pgsA* in wild type. As shown in **Figure 2C**, both *CrPGP1* and *CrPGP2* were expressed, whose levels were fivefold and eightfold higher than that of *pgsA*. Thus, under the *psbA2* promoter control, both *CrPGP1* and *CrPGP2* were expressed in *pgsA CrPGP1* and *pgsA CrPGP2*.

### Complementation of *Synechocystis* sp. PCC 6803 *pgsA* Mutant with *CrPGPs*

**FIGURE 3:**
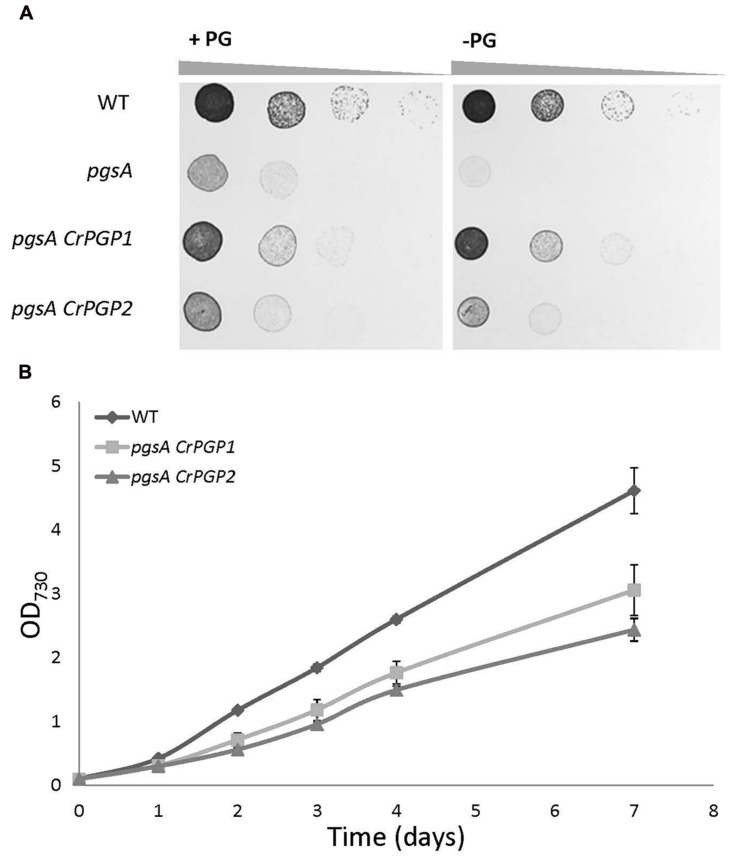
**Growth of *pgsA CrPGP1* or *pgsA CrPGP2*. (A)** Growth of the wild type, *pgsA*, *pgsA CrPGP1*, or *pgsA CrPGP2* on solid BG-11 media with or without PG supplementation. Spotting involved serial 10-fold dilution from left to right starting at OD_730_ 0.05, with 5 μl each spotted onto a BG-11 agar plate with or without 20 μM PG and incubation under 50∼60 μmol photons m^-2^ s^-1^ for 5 days at 30°C. Images are representative of three biological replicates. **(B)** Growth profile of the wild type, *pgsA CrPGP1* or *pgsA CrPGP2* in liquid BG-11 media. Growth was initiated at OD_730_ 0.1 by stirring culture at 180 rpm, 50∼60 μmol photons m^-2^ s^-1^ at 30°C. Data are mean ± SD from three biological replicates.

Previous study showed that the *pgsA* mutant of *Synechocystis* sp. PCC 6803 requires exogenous supplementation of PG for growth (Hagio et al., 2000), so we compared the growth of the wild type, *pgsA*, *pgsA CrPGP1*, and *pgsA CrPGP2* on solid BG-11 media with or without 20 μM PG. The wild-type cells grew similarly in the presence or absence of PG, whereas the *pgsA* mutant showed rescued growth only with PG (**Figure 3A**). The *pgsA CrPGP1* and *pgsA CrPGP2* showed rescued growth even in the absence of PG, which suggests that *CrPGP1* and *CrPGP2* are functional PGPS to rescue the growth defect of *pgsA*. To further investigate the growth profiles of *pgsA CrPGP1* or *pgsA CrPGP2*, we monitored the growth in liquid BG-11 media without PG. Although the growth rate was significantly restored in the transgenic strains, it was inferior to that of the wild type, with the *pgsA CrPGP1* showed slightly better growth than *pgsA CrPGP2* (**Figure 3B**). Therefore, *CrPGP1* and *CrPGP2* could functionally complement the lethal phenotype of the *pgsA* mutant, although their growth remain slightly retarded as compared with the wild type.

### Lipid Composition of *pgsA CrPGP1* and *pgsA CrPGP2*

**FIGURE 4:**
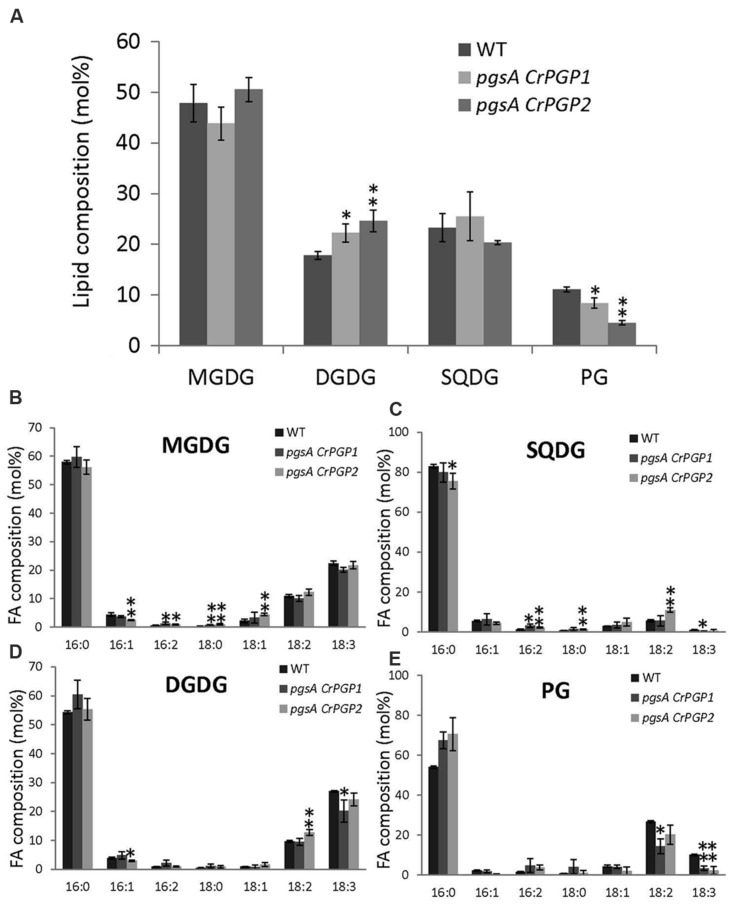
**Polar glycerolipid composition **(A)** and fatty acid composition of **(B)** MGDG; **(C)** SQDG; **(D)** DGDG; and **(E)** PG of the wild type, *pgsA CrPGP1* and *pgsA CrPGP2*.** Data are mean ± SD from three biological replicates. Asterisks show significance (^∗^*p* < 0.05, ^∗∗^*p* < 0.01) from the wild type. Unsaturated fatty acids detected in all lipid classes contain only *cis* double bonds.

To examine whether the levels of PG and other polar glycerolipids are restored in *pgsA CrPGP1* or *pgsA CrPGP2*, we analyzed the composition of membrane lipid classes and their fatty acid composition. PG composition in the wild type was 11 mol% but was 8 and 5 mol% in *pgsA CrPGP1* and *pgsA CrPGP2*, respectively (**Figure 4A**). Moreover, composition of DGDG was slightly higher in the transgenic strains than the wild type. Next, we analyzed the fatty acid composition of these lipid classes (**Figures 4B–E**). The fatty acid composition of MGDG, DGDG, and SQDG was fairly similar among the three strains, but that of PG was markedly decreased in 18:3 and 18:2 composition and increased in 16:0 in *pgsA CrPGP1* and *pgsA CrPGP2* (**Figure 4E**). Hence, *CrPGP1* and *CrPGP2* produced a significant level of PG in *pgsA*, but the level was slightly lower and fatty acid composition differed from that of the wild type.

### Pigment Content and Oxygen Evolution Activity of *pgsA CrPGP1* and *pgsA CrPGP2*

**FIGURE 5:**
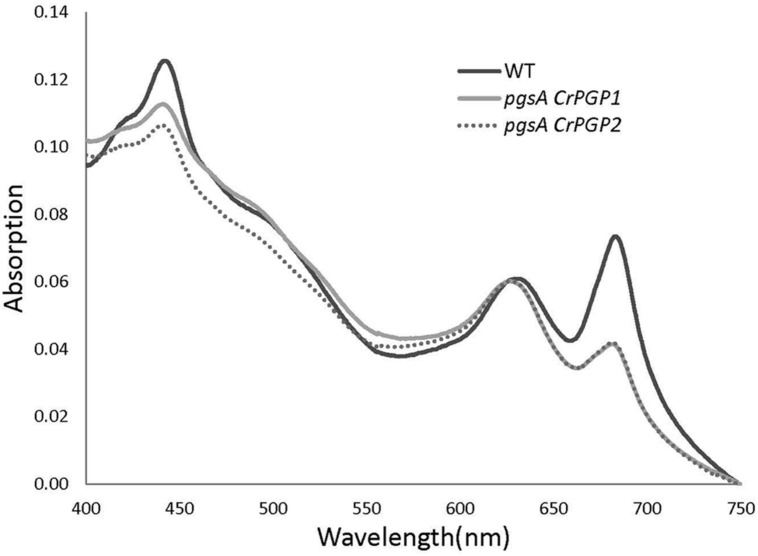
**Absorption spectra of wild type, *pgsA CrPGP1* and *pgsA CrPGP2* in BG-11 medium.** The spectra were measured with whole cells (OD_730_ ≈ 0.1) and normalized at 625 nm, the maximum absorption of phycobiliproteins.

Previous studies have shown that PG deficiency reduces Chl content in *Synechocystis* sp. PCC 6803 and other cyanobacteria (Gombos et al., 2002; Wu et al., 2006; Bogos et al., 2010). To assess whether the expression of *CrPGPs* can restore the Chl content in *pgsA*, we examined absorption spectra of pigments in the whole cells of the wild type, *pgsA CrPGP1* and *pgsA CrPGP2* (**Figure 5**). The spectra was normalized at 625 nm, the maximum absorption of phycobiliproteins. Absorptions at ∼683 and ∼441 nm by Chl were lower for both *pgsA CrPGP1* and *pgsA CrPGP2* than the wild type. Indeed, the cellular Chl content in both complemented strains was decreased to 63% and 68% of the wild-type level, respectively (**Table 1**). To elucidate whether *CrPGPs* can restore the photosynthetic activity in *pgsA*, we compared the net oxygen evolution rate in complemented strains and the wild type. On a Chl content basis, oxygen evolution activities in *pgsA CrPGP1* and *pgsA CrPGP2* cells were 57 and 61% less than wild-type activities, respectively, which corresponded to 73 and 73.5% less than the wild type on a cell density basis. Therefore, the introduction of *CrPGP1* or *CrPGP2* can partially but not fully complement the loss of *pgsA* in *Synechocystis* sp. PCC 6803 in terms of photosynthetic activity as well as Chl accumulation.

**Table 1:** Photosynthetic oxygen-evolving activities and Chl contents in intact cells of wild type, *pgsA CrPGP1*, and *pgsA CrPGP2* of *Synechocystis* sp. PCC 6803.

Strains	μg Chl/OD_730_	Net electron transfer (μmol O_2_ mg Chl^-1^ h^-1^)	Net electron transfer (μmol O_2_ OD_730_^-1^ h^-1^)
Wild type	0.62 ± 0.05	320 ± 20	20 ± 1.4
*pgsA CrPGP1*	0.39 ± 0.03	140 ± 30	5.4 ± 1.1
*pgsA CrPGP2*	0.42 ± 0.03	130 ± 20	5.3 ± 0.7

*Intact cells were treated for 2 min in darkness, and oxygen evolution was measured at saturating light. The temperature of measurements was 30°C. Data are mean ± SD from three independent measurements*.

### Electron Transfer Kinetics in the Acceptor and Donor Side of PSII in *pgsA CrPGP1* and *pgsA CrPGP2*

**FIGURE 6:**
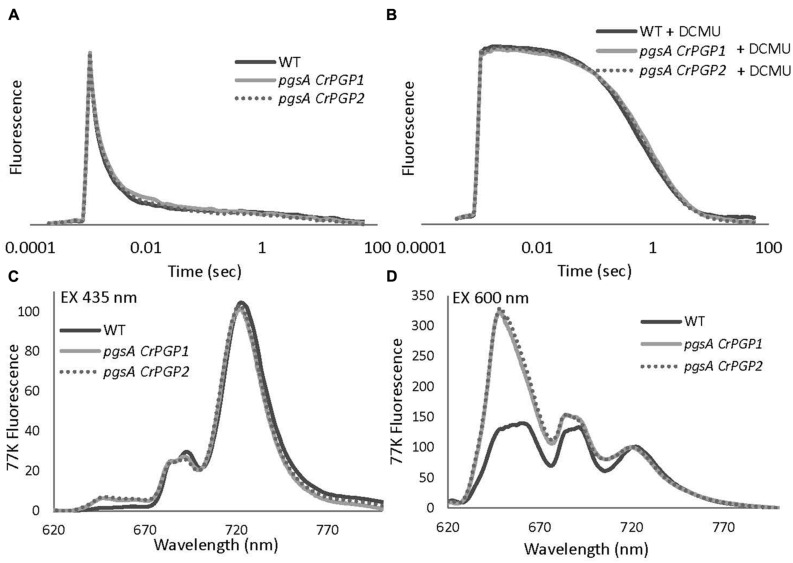
**Relaxation of flash-induced Chl fluorescence yield **(A,B)** and 77 K fluorescence emission spectra **(C,D)** of wild type, *pgsA CrPGP1* and *pgsA CrPGP2* mutant cells in BG-11 medium.** The spectra resulted from a single flash excitation without **(A)** or with **(B)** 10 μM DCMU. Data are the mean from three independent experiments. In each case, Chl concentrations were adjusted to 5 μg mL^-1^. Samples were incubated in darkness for 10 min before DCMU was added to the final concentration of 10 μM. The levels of *F*_o_ and *F*_m_ were normalized. **(C,D)** 77 K fluorescence emission spectra from the wild type, *pgsA CrPGP1* and *pgsA CrPGP2* mutant cells were measured under 435 nm **(C)** or 600 nm **(D)** excitation. Data are the mean from three independent experiments. Spectra were normalized at 720 nm to 100. In each case, Chl concentrations were adjusted to 10 μg mL^-1^.

The low oxygen evolution activities in the *pgsA CrPGP1* or *pgsA CrPGP2* imply impaired photosynthetic electron transport in these strains. To characterize the functionality of electron transfer within PSII in the complemented strains, we evaluated the reoxidation kinetics of Q_A_, the primary electron acceptor plastoquinone (PQ) of PSII, by analyzing the decay of Chl fluorescence after a single saturating flush. The kinetics of accepter-side electron transfer from Q_A_^-^ to the PQ pool was evaluated without DCMU (**Figure 6A**), and the kinetics from Q_A_^-^ to the donor-side components were evaluated with DCMU, which inhibits the electron transfer from Q_A_ to Q_B_ and causes charge recombination between Q_A_^-^ and the oxidizing-side components (**Figure 6B**). In both *pgsA CrPGP1* and *pgsA CrPGP2* cells, the fluorescence decay kinetics were almost identical to those in the wild type without or with DCMU. Thus, both complemented strains may assemble the functional PSII complex as in the wild type.

### Chl Fluorescence Emission Spectra at 77 K in *pgsA CrPGP1* and *pgsA CrPGP2*

The core-antenna complexes of PSI and PSII are largely disordered in the PG-deficient *pgsA* mutant. To evaluate whether *CrPGPs* restore PS-antenna complexes in *pgsA*, we examined emission spectra of Chl fluorescence at 77 K. In both wild-type and mutant cells, the preferential excitation of Chl at 435 nm resulted in four emission peaks, at ∼647, ∼681, ∼691, and ∼720 nm (**Figure 6C**). The emission peak at ∼647 nm can be attributed to phycocyanin (Rakhimberdieva et al., 2007); peaks at ∼681 nm and ∼691 nm primarily originate from CP43 and CP47, which are functionally coupled to the PSII reaction center, respectively; and the peak at ∼720 nm originates from PSI. For *pgsA CrPGP1* and *pgsA CrPGP2* cells, we found only a slight decrease in emission peak at ∼691 nm as compared with the wild type, which suggests a small change in the PSII complex in these complemented strains. By contrast, the emission from phycocyanin at 647 nm was slightly increased in the complemented strains. To examine the energy coupling between phycobilisomes and PS cores in the complemented lines, phycobilins were preferentially excited at 600 nm at 77 K (**Figure 6D**). Both *pgsA CrPGP1* and *pgsA CrPGP2* cells showed prominent emissions at ∼647 and ∼681 nm as compared with wild-type cells. The emission at ∼647 nm originates from phycocyanin, whereas that at ∼681 nm is contributed by both PSII Chl and terminal phycobilin emitters. The strong enhancement of the emission peaks at ∼647 and ∼681 nm in the complemented lines suggests that the energy transfer from phycobilisomes to the PSII reaction center is uncoupled in these cells.

### Gene Expression Profiles of *CrPGP1* and *CrPGP2* under Nutrient-Limited *C. reinhardtii*

**FIGURE 7:**
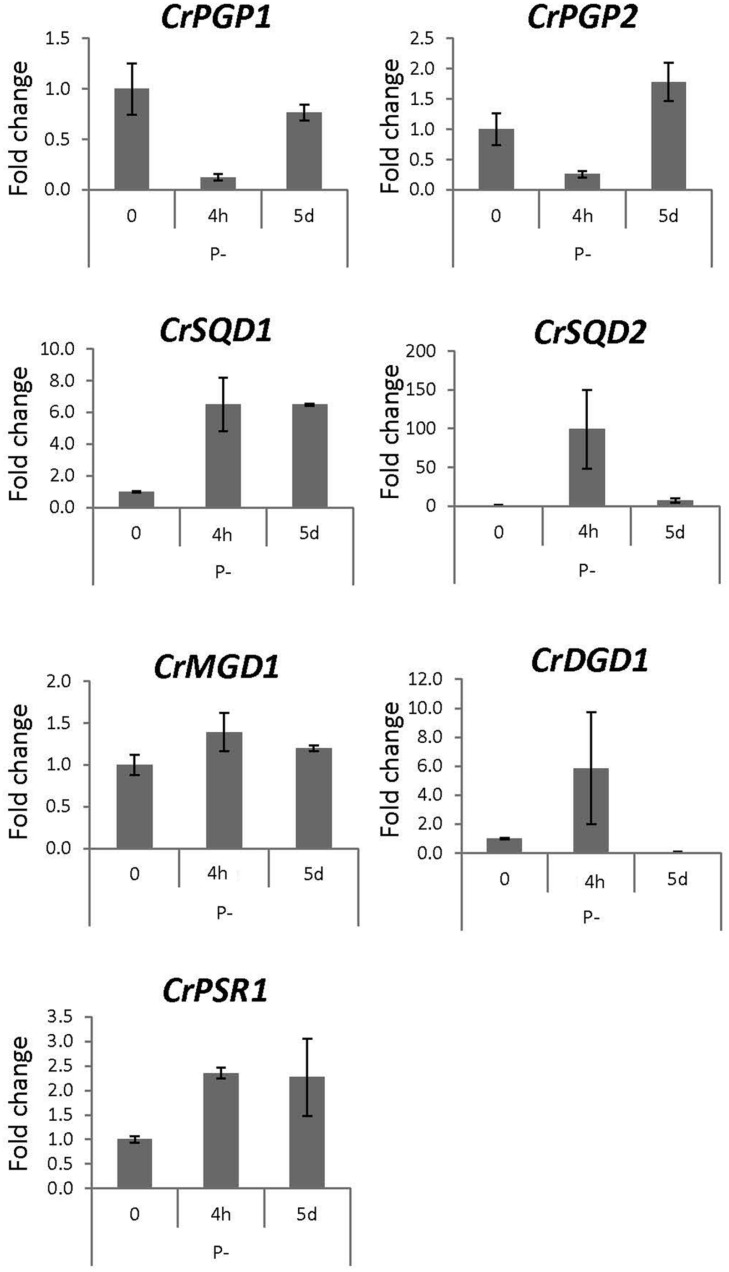
**Gene expression profile of enzymes for plastidial polar glycerolipid biosynthesis under phosphorus starvation**. Wild-type *C. reinhardtii* strain CC-4351 grown in TAP media was transferred to the media devoid of phosphate and harvested at 4 h and 5 days. Total RNA was extracted to synthesize cDNA for qRT-PCR analysis. Gene expression was normalized to that of *CBLP*. Data were averaged by three technical replicates in the same run and two biological replicates in separate runs. *SQD1*, *UDP-sulfoquinovose synthase*; *SQD2*, *sulfoquinovosyldiacylglycerol synthase*; *MGD1*, *MGDG synthase1*; *DGD1*, *DGDG synthase1*; *PSR1*, *phosphorus starvation response1*.

**FIGURE 8:**
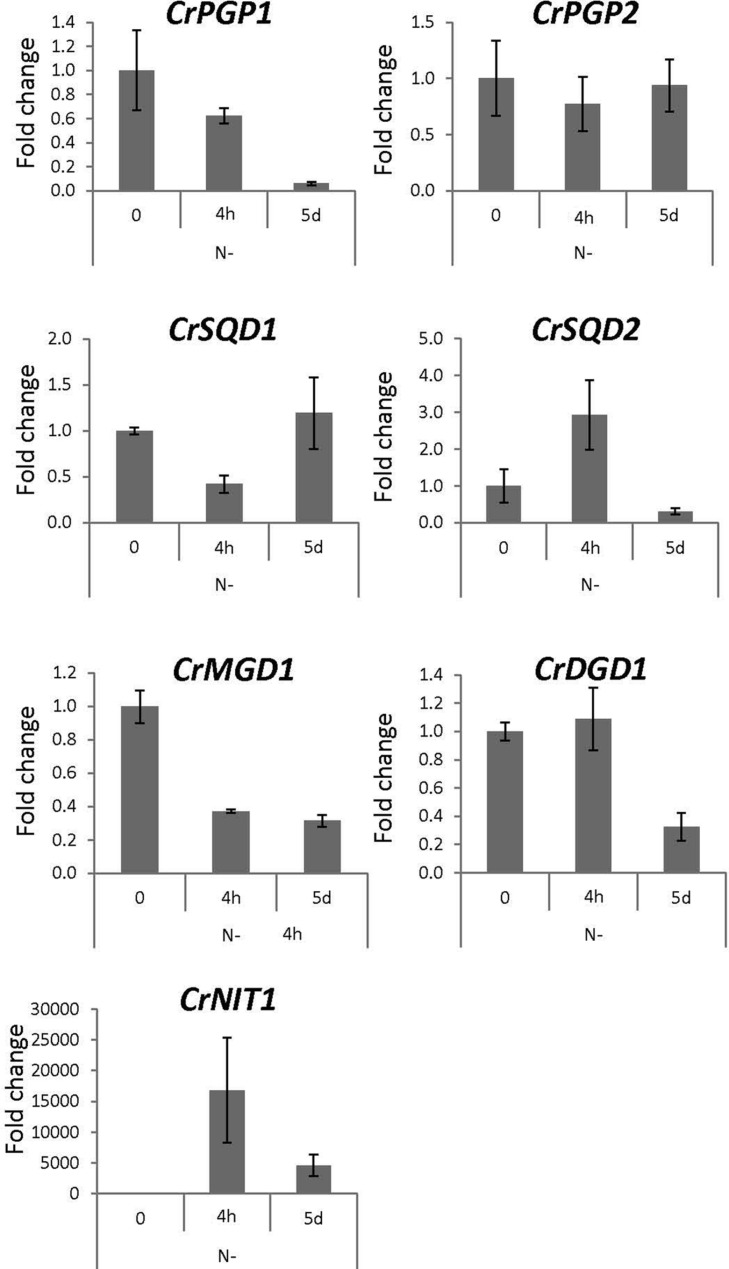
**Gene expression profile of enzymes for plastidial polar glycerolipid biosynthesis under nitrogen starvation.** *NIT1, nitrate reductase 1*. See legend of **Figure 7** for sampling conditions, experimental methods, and abbreviations.

To obtain physiological insight into the role of CrPGP1 and CrPGP2, we analyzed gene expression profiles of *CrPGP1* and *CrPGP2* together with related genes for glycerolipid metabolism during phosphorus or nitrogen starvation. During phosphorus starvation, significant portion of phospholipids is replaced by non-phosphorus galactolipid DGDG, termed membrane lipid remodeling (Nakamura, 2013). Moreover, SQDG is increased and compensates for the decreased PG and maintains the total amount of anionic lipid classes (Riekhof et al., 2003). However, gene expression profiles of enzymes related to this metabolic change were not studied previously. Here, we took two time points after switching *C. reinhardtii* to phosphorus starvation: 4 h for the early response and 5 days for the late response (**Figure 7**). *CrPSR1* is a marker gene for phosphorus starvation response (Wykoff et al., 1999). Both *CrPGP1* and *CrPGP2* showed a transient decrease at 4 h but recovered at 5 days. In contrast, the expression of *CrSQD1* and *CrSQD2*, which are required for SQDG biosynthesis, was induced at 4 h. Moreover, the gene expression of *CrDGD1* was increased but that of *CrMGD1* was not, which supports an increase in DGDG but not MGDG upon phosphorus starvation. These results showed that expression profiles of *CrPGP1* and *CrPGP2*, as well as other related genes examined in **Figure 7**, are altered under phosphorus starvation. Next, we examined nitrogen starvation using *CrNIT1* as a marker gene (Hipkin et al., 1980). This condition severely affects photosynthesis but induces accumulation of triacylglycerol (Wang et al., 2009). As shown in **Figure 8**, *CrPGP1* but not *CrPGP2* showed decreased gene expression under nitrogen starvation. *CrSQD1* and *CrSQD2* showed transient changes in gene expression level, and *CrMGD1* and *CrDGD1* both decreased the gene expression level at different time points. Thus, *CrPGP1* and *CrPGP2* showed differential gene expression profiles under phosphorus starvation and nitrogen starvation conditions.

## Discussion

Phosphatidylglycerol synthesis is critical to maintain the stability of membrane protein complexes in photosynthesis components and in the respiratory electron transfer chain (Hagio et al., 2000; Ostrander et al., 2001; Domonkos et al., 2004; Hasan et al., 2013). This study isolated a pair of *PGPS* genes, *CrPGP1* and *CrPGP2*, in a model eukaryotic green microalga *C. reinhardtii*. The molecular function of these newly isolated PGPs was revealed by transforming them into the *pgsA* mutant of *Synechocystis* sp. PCC 6803, a representative cyanobacterium. Moreover, gene expression profiles of *CrPGP1* and *CrPGP2* were investigated in *C. reinhardtii* under phosphorus or nitrogen starvation. Strong responses of *CrPGPs*, particularly *CrPGP1*, to these conditions imply their involvements in membrane lipid remodeling in re sponse to nutrient starvation. The results of cell growth, lipid analysis, and photosynthetic parameters in *pgsA CrPGP1* and *pgsA CrPGP2* showed distinct and overlapping function of PGPS between cyanobacteria and eukaryotic algae.

The heterologous complementation of the *pgsA* mutant phenotype by *CrPGP1* or *CrPGP2* supports that they encode a functional PGPS of *C. reinhardtii* (**Figures 3A,B**). However, the slower growth profile in *pgsA CrPGP1* or *pgsA CrPGP2* than the wild type under liquid culture suggests that *CrPGPs* cannot fully rescue the defect of *pgsA* mutant (**Figure 3B**). Our lipid analysis agrees with this idea: as compared with the wild type, *pgsA CrPGP1* and *pgsA CrPGP2* showed reduced PG contents by 73 and 45%, respectively. Although the expression level of *CrPGP2* was higher than *CrPGP1* (**Figure 2C**), expression of the CrPGP1 protein complemented the *pgsA* mutant phenotype more effectively than that of the CrPGP2. The protein sequence similarity with PgsA was slightly higher for CrPGP1 (63.0%) than CrPGP2 (62.6%). Moreover, *in silico* prediction of possible subcellular localization by ChloroP (Emanuelsson et al., 1999), TargetP (Emanuelsson et al., 2000), or PledAgro (Tardif et al., 2012) suggests that CrPGP1 may be localized at the chloroplasts or mitochondria whereas CrPGP2 may be localized somewhere other than the chloroplasts, the mitochondria or the secretory pathway. Indeed, longer N-terminal sequence was found in CrPGP1 (**Figure 1A**). Because the different subcellular localization was shown between AtPGP1 and AtPGP2 (Babiychuk et al., 2003; Tanoue et al., 2014), it is possible that CrPGP1 and CrPGP2 also have different subcellular localizations. It is possible that CrPGP1 is functionally more similar to PgsA because of the chloroplast localization. The fatty acid composition of PG showed reduced composition of 18:3 (**Figure 4E**). Because 18:3 in PG is produced by the fatty acid desaturase that desaturates acyl groups of PG, thus it is possible that PG produced by CrPGP1 and CrPGP2 may not be properly desaturated in *pgsA*, which highlights the different enzymatic features between PGP of *Synechocystis* sp. PCC 6803 and *C. reinhardtii*.

Many PG molecules are found in the PSII complex (Sakurai et al., 2006) and are present near the reaction center (Guskov et al., 2009; Umena et al., 2011). In fact, PG is indispensable for both donor- and acceptor-side activities of PSII (Hagio et al., 2000; Sato et al., 2000; Gombos et al., 2002; Sakurai et al., 2003, 2007a). Our data in **Figure 6** reveals that the expression of *CrPGPs* in *pgsA* sufficiently restored electron transfer activities of PSII to the wild-type level, although the PG levels and fatty acid composition in the complemented strains differed somewhat from those in the wild type (**Figure 4**). Thus, CrPGP activities would be enough to supply PG molecules to the PSII complex in the *pgsA* background. Meanwhile, the oxygen evolution activity in *pgsA* was not fully recovered by introducing the *CrPGPs* and remained at low levels (**Table 1**). Because phycobilisomes, which are not present in green algae including *Chlamydomonas*, may be energetically uncoupled with the PSII reaction center in the *pgsA* mutants harboring *CrPGPs* (**Figure 6D**), loss of energy transfer from phycobilisomes to the PSII reaction center in part likely reduces net photosynthesis activities in the complemented strains. The PG-deficient *pgsA* mutant showed energetic uncoupling between phycobilisomes and the PSII reaction center and not fully restored by the supplementation of PG (Sakurai et al., 2007a). Thus, *in situ* PG synthesis by native PgsA may be important for assembly of phycobilisomes with the PSII complex in *Synechocystis* sp. PCC 6803.

The expression of *CrPGP1* and *CrPGP2*, as well as the other genes showed differential profiles under two different nutrient starvation conditions (**Figures 7** and **8**). Most genes involved in glycoglycerolipid biosynthesis (*CrSQD1*, *CrSQD2*, and *CrDGD1*) showed rapid upregulation upon phosphorus starvation, whereas expression of *CrPGP1* and *CrPGP2* was decreased at 4 h after phosphorus starvation. This profile is in agreement with an increase in DGDG and SQDG levels and a decrease in PG levels under phosphorus starvation (Riekhof et al., 2003). However, the reduced expression of *CrPGP1* and *CrPGP2* was recovered at 5 days after phosphorus starvation. In addition to the membrane lipid remodeling, *C. reinhardtii* cells induce triacylglycerol biosynthesis by long-term phosphorus starvation with retaining thylakoid membrane networks (Iwai et al., 2014) and photosynthetic activity (Wykoff et al., 1998). Thus, the recovered expression of *CrPGP1* and *CrPGP2* may function to keep proper balance of lipid composition of thylakoid membranes and maintain photosynthetic activity even under long-term phosphorus starvation, at which triacylglycerol is produced possibly for an energy storage. Meanwhile, expression of most of the genes in **Figure 8** was decreased in response to nitrogen starvation. These changes agree with the actual decrease in MGDG, DGDG, SQDG, and PG, degradation of thylakoid membranes, and loss of photosynthetic activity under nitrogen starvation (Siaut et al., 2011; Iwai et al., 2014; Sakurai et al., 2014). Notably, *CrPGP1* but not *CrPGP2* showed decreased expression, whose profile was similar to that of *CrMGD1* upon nitrogen starvation. This suggests distinct roles between CrPGP1 and CrPGP2, which was also presumed by possible differences in subcellular localization and enzyme property of these isozymes. Differential expression profiles of some of lipid biosynthesis genes were previously observed. For example, the gene expression levels of *CrSQD1* showed partial recovery at later time points, *CrSQD2* expression showed fluctuation while decreasing, and *CrMGD1* expression showed faster decrease than that of *CrDGD1* upon nitrogen starvation (Boyle et al., 2012). These data are generally consistent with our results in **Figure 8**, although some differences were observed probably because of differences in growth conditions and/or time point analyzed. The distinct expression profiles among genes involved in membrane lipid biosynthesis including *CrPGP1* and *CrPGP2* may allow *C. reinhardtii* cells to fine-tune lipid metabolism in response to various nutrient conditions.

## Conclusion

Our results reveal that both *CrPGP1* and *CrPGP2* encode functional PGPS, which can contribute to PG production and the formation of photosynthetic complexes in the PG-deficient *Synechocystis pgsA* mutant. Further analyses are required to elucidate the roles of CrPGP1 and CrPGP2 in lipid metabolism and photosynthesis in *C. reinhardtii*.

## Conflict of Interest Statement

The authors declare that the research was conducted in the absence of any commercial or financial relationships that could be construed as a potential conflict of interest.

## Abbreviations

Chl: chlorophyll
DAG: *sn*-1,2-diacylglycerol
DCMU: 3-(3,4-dichlorophenyl)-1,1-dimethylurea
DGDG: digalactosyldiacylglycerol
MGDG: monogalactosyldiacylglycerol
MGlcDG: monoglucosyldiacylglycerol
PG: phosphatidylglycerol
PGPS: phosphatidylglycerophosphate synthase
PS: photosystem
SQDG: sulfoquinovosyldiacylglycerol
