# Supplementary material for: Characterization of Chlamydomonas reinhardtii phosphatidylglycerophosphate synthase in Synechocystis sp. PCC 6803
Source: Front Microbiol. 2015 Aug 24;6:842. doi: 10.3389/fmicb.2015.00842 (PMC4547039; doi:10.3389/fmicb.2015.00842)
Supplement: Supplementary file 1 [file Table_1.DOCX]

***Supplementary Material***

**Characterization of *Chlamydomonas reinhardtii* phosphatidylglycerophosphate synthase in *Synechocystis* sp. PCC 6803**

Chun-Hsien Hung^1^, Kaichiro Endo^2^, Koichi Kobayashi^2^, Yuki Nakamura^1,3*^ and Hajime Wada^2,4^

^1^Institute of Plant and Microbial Biology, Academia Sinica, Taipei, Taiwan.

^2^Department of Life Sciences, Graduate School of Arts and Sciences, The University of Tokyo, Japan.

^3^PRESTO , Japan Science and Technology Agency, Saitama, Japan.

^4^CREST, Japan Science and Technology Agency, Saitama, Japan.

*Corresponding: Yuki Nakamura, Institute of Plant and Microbial Biology, Academia Sinica, 128 sec.2 Academia Rd., Nankang, Taipei 11529, Taiwan. [nakamura@gate.sinica.edu.tw](mailto:nakamura@gate.sinica.edu.tw)

**Supplementary Tables**

| **Supplemental Table 1 List of strains used in this study.** | | | | |
| --- | --- | --- | --- | --- |
| **Strains** | **Genotype** | **Encoded gene** | **Selection marker** | **Source** |
| *Synechocystis* sp. PCC 6803 | Wild type | - | - | - |
| *pgsA* | *pgsA*::Km^R^ | *pgsA*::Km^R^ | Km^R^ | (Hagio et al. 2000) |
| CHS004 | ∆*pgsA* *slr2031*∆::*CrPGP1* | *CrPGP1* (Cre03.g162601) | Cm^R^ | This work |
| CHS005 | ∆*pgsA slr2031*∆::*CrPGP2* | *CrPGP2* (Cre02.g095106) | Cm^R^ | This work |
| CC-4351 | cw15 arg7-8 mt+ | *-* | - | Chlamydomonas Resource Center |
| CC-503 | cw92 mt+ | *-* | - | Chlamydomonas Resource Center |
| CHC041 | cw15 arg7-8 mt+ | *pChlamiRNA2* | *ARG7* | This work |
