# Supplementary material for: Characterization of Chlamydomonas reinhardtii phosphatidylglycerophosphate synthase in Synechocystis sp. PCC 6803
Source: Front Microbiol. 2015 Aug 24;6:842. doi: 10.3389/fmicb.2015.00842 (PMC4547039; doi:10.3389/fmicb.2015.00842)
Supplement: Supplementary file 2 [file Table_2.DOCX]

| **Supplemental Table 2 List of plasmids used in this study.** | | | | | |
| --- | --- | --- | --- | --- | --- |
| **Plasmids** | **Encoded gene** | **Promoter** | **Vector** | **Selection marker** | **Source** |
| pTCP2031V | - | *psbA2* | **-** | Amp^R^ / Cm^R^ | (Satoh et al. 2001) |
| pENTR/D-TOPO | - | - | - | Km^R^ | Invitrogen |
| pCH067 | *CrPGP1* (Cre03.g162601) | - | pENTR/D-TOPO | Km^R^ | This work |
| pCH068 | *CrPGP2* (Cre02.g095106) | - | pENTR/D-TOPO | Km^R^ | This work |
| pCH167 | *CrPGP1* (Cre03.g162601) | *psbA2* | pTCP2031V | Amp^R^ / Cm^R^ | This work |
| pCH160 | *CrPGP2* (Cre02.g095106) | *psbA2* | pTCP2031V | Amp^R^ / Cm^R^ | This work |
