# Supplementary material for: Characterization of Chlamydomonas reinhardtii phosphatidylglycerophosphate synthase in Synechocystis sp. PCC 6803
Source: Front Microbiol. 2015 Aug 24;6:842. doi: 10.3389/fmicb.2015.00842 (PMC4547039; doi:10.3389/fmicb.2015.00842)
Supplement: Supplementary file 3 [file Table_3.DOCX]

| **Supplemental Table 3 List of oligonucleotides used in this study.** | |
| --- | --- |
| **Name** | **Sequences (5’ to 3’)** |
| CH223 | CACCATGGCCATGTTAGGAAAGGTCTGCT |
| CH224 | TCACATCCTGGCCATGAACCTCCAC |
| CH225 | CACCATGGTGGAAGCTACGGAGTCTGCAG |
| CH226 | TCAGTGGTGCTTCTTGGCCTCGGGG |
| CH772 | TTAGGTACCGGAATTCCATATGGAATTCCATGGCCATGTTAGGAAAGGTCTGC |
| CH773 | TAAAAGCTTTAGGTTAACTCACATCCTGGCCATGAACCTCCA |
| CH774 | GGAATTCCATATGGAATTCCATGGTGGAAGCTACGGAGTCTGCA |
| CH775 | TAGGTTAACTCAGTGGTGCTTCTTGGCCTCGGG |
| CH784 | GGAAGGCAACCATCTGGGACTGAT |
| CH785 | CAGGGATTTGGGCAAACTAGCTCA |
| CH888 | ATCGACCAGGCTTGCTCGTA |
| CH889 | GTCAATGTTGTCGATGTCGAAGA |
| CH890 | AATTGTTCCCGCGTTCCT |
| CH891 | CCGCTCTTGTTGACATTGC |
| CH919 | CAGCTACGCTCATGGATTGA |
| CH920 | TGCACTTGCATGCCCATTC |
| CH947 | TGAAAAGGGTAAGGGTGCAAAG |
| CH948 | AAATTCCTCAAGCGGTTCCA |
| CH953 | CCCAACGTGCTCACCTTCTT |
| CH954 | TTCTCGCCAGGTATCCATCAA |
| CH982 | CATGGGGAAGTTTGCTGGTTCAAT |
| CH1000 | CGGCCCACTTCCTCTTGGTGACTC |
| CH1060 | ACTACGAGTCATGGCAACAATCC |
| CH1028 | TTTATTGGCTTTACCCCTGCTATT |
| CH1029 | CCCAACTCCGTCACCTGATT |
| CH1063 | TGGCGAACAGCACGTTAATG |
| CH1064 | AAGCGCGTTTGCATCTTT |
| CH1065 | CACACCCGGTAGCGTGTAG |
| CH1066 | CCGACCTCTCCAGCAA |
| CH1067 | TGCTGTCATTGAGCAAACC |
| CH1072 | CTCGGACGTTATCATCGCATAC |
| CH1073 | TTCTGCGACTCCGTGTCCAT |
| CH1076 | TCATCCACTGCCTGTGCTTCT |
| CH1077 | GGCCTTCTTGCTGGTGATGT |
